# A Case of Tuberculous Meningitis with Paradoxical Response in a 14-Year-Old Boy

**DOI:** 10.1155/2016/5875628

**Published:** 2016-10-11

**Authors:** Murat Özer, Yasemin Özsürekci, Ali Bülent Cengiz, Nagehan Emiralioğlu, Deniz Doğru, Kader Karlı Oğuz, Onur Akça, Özgür Özkayar

**Affiliations:** ^1^Department of Pediatrics, Hacettepe University Faculty of Medicine, Ankara, Turkey; ^2^Pediatric Infectious Diseases, Hacettepe University Faculty of Medicine, Ankara, Turkey; ^3^Pediatric Chest Diseases, Hacettepe University Faculty of Medicine, Ankara, Turkey; ^4^Department of Radiology, Hacettepe University Faculty of Medicine, Ankara, Turkey; ^5^Department of Pathology, Hacettepe University Faculty of Medicine, Ankara, Turkey

## Abstract

A clinical or radiological worsening of already existing lesions or an emergence of new lesions after beginning treatment in patients with tuberculosis (TB) is referred to as the paradoxical response. This has aroused suspicion regarding the accuracy of diagnosis, the possibilities of treatment failure, or the presence of another underlying disease, and thus it is an important topic for clinicians to understand. In this article, the development of a paradox reaction in a 14-year-old male patient diagnosed with and treated for tuberculosis meningitis is reported. This pediatric patient with a healthy immune system is treated with steroids successfully and reported to elucidate the importance of managing the paradox of TB progression in spite of the appropriate anti-TB medications.

## 1. Introduction

In tuberculosis (TB) patients, the clinical or radiological worsening of already existing lesions or new lesions appearing after treatment is called a paradoxical response [1]. Paradoxical responses are especially seen in lungs, lymph nodes, and the central nervous system affected by TB [2]. There is scarce data about the incidence of paradoxical reactions in HIV-negative children in literature. In 115 HIV-negative children with pulmonary and extrapulmonary TB, 12 patients (10%) developed a paradoxical reaction [3]. Therefore, awareness of clinicians about the clinical nature of this situation is important, particularly in patients with treatment failure or underlying diseases [2]. It poses serious problems in the management of central nervous system (CNS) TB. There is always a question of development of drug resistant TB when there is worsening of the patient and often paradoxical reaction leads to inappropriate increment or addition of more toxic newer antitubercular drugs with several adverse consequences [4]. Herein, the case of a child with TB meningitis with a normal immune system, who was observed to have paradoxical responses, was reported to emphasize this situation and increase clinicians' awareness.

## 2. Case Report

Two months before being admitted to our hospital, a 14-year-old male patient was admitted to another hospital because of headache, vomiting, and fever. An emerging hemiparesis persisted on the left side of the patient. When his family history was questioned, the patient's father was treated 1.5 years ago for lung TB (caused by resistant* Mycobacterium tuberculosis* bacilli); however, it was learned that prophylaxis was not given to the patient. Tuberculosis meningitis was diagnosed according to physical examination and laboratory findings and radiological findings in magnetic resonance imaging (MRI) of brain. Anti-TB treatments (isoniazid, rifampicin, ethambutol, and pyrazinamide) and 0.5 mg/kg of prednisolone were started for CNS TB. Complaints improved with the treatment, and the prednisolone was decreased gradually. He was discharged at 38th day of the treatment with a plan of follow-up outpatient clinic visits.

**Figure 1:**
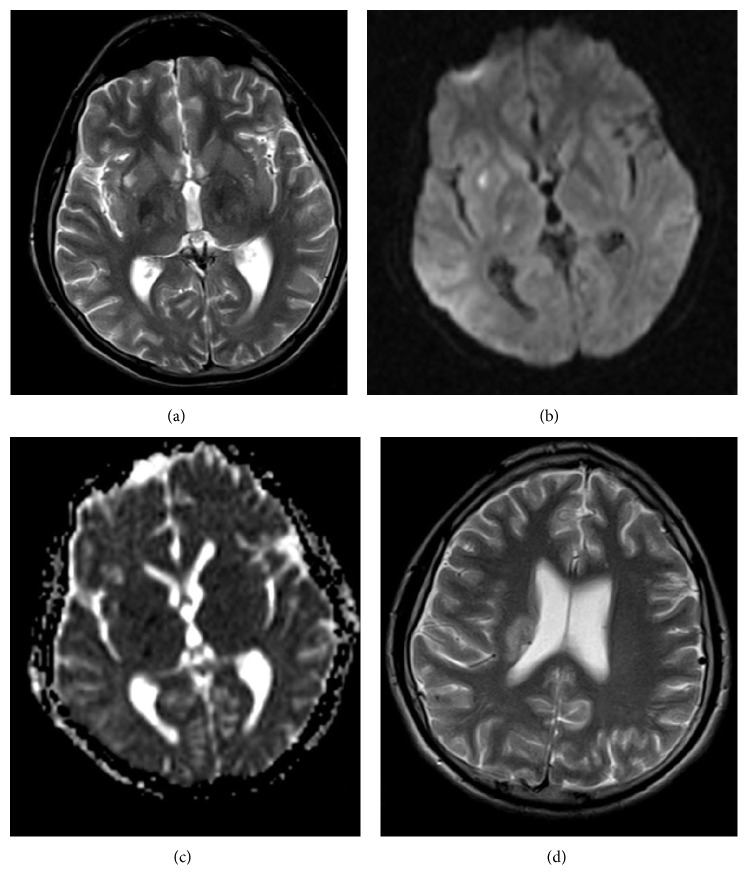
Instant diagnosis T2A (a), diffusion image (b = 1000 s/mm^2^) (b), ADC (c) map, and T2A image 15 days after diagnosis (d). Acute ischemic lesions in the right thalamus, globus pallidus, and lateral putamen (b, c), increases of bilateral hypothalamic T2 intensity (a), and corona radiata intensity increment after 15 days (d). After IVKM there was no inclusion of a parenchymal contrast.

**Figure 2:**
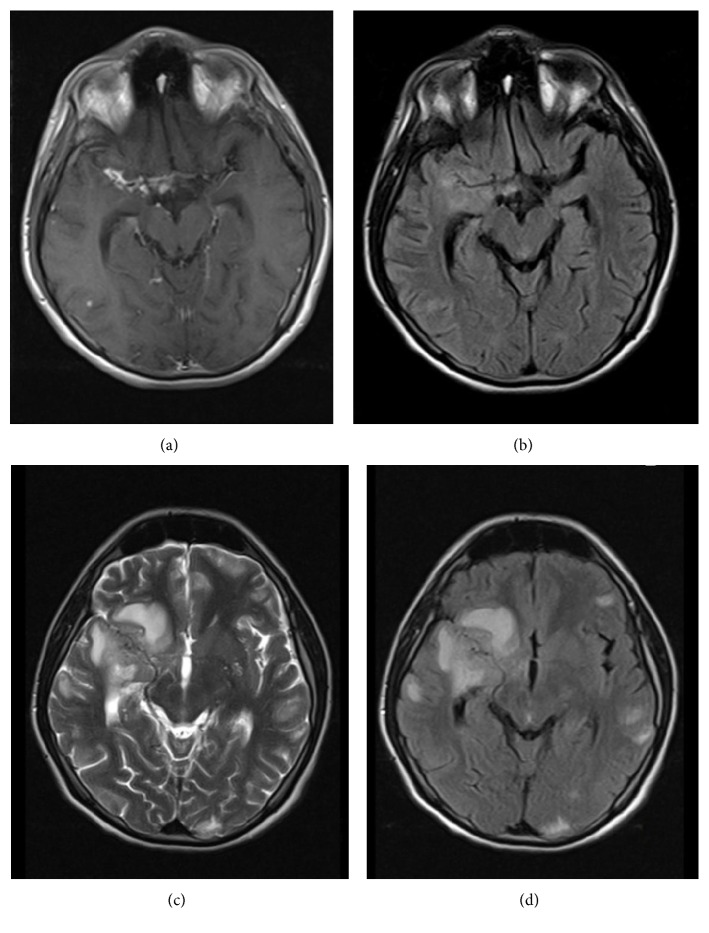
A contrast of an initial diagnosis against its progression after one month T1A (a), FLAIR (b), a diagnosis 45 days later with T2A (c), and FLAIR (d) images. A meningeal contrast is involved and a thickening compatible with a diagnosis of basal meningitis is visible in the neighborhood of the right sylvian fissure and basal cisterns (a) and increments of parenchymal T2 intensity in that region (b). Instead of antituberculosis treatments working, on the forty-fifth day mark, parenchymal lesions have been proven to grow and new lesions have appeared (c, d).

**Figure 3:**
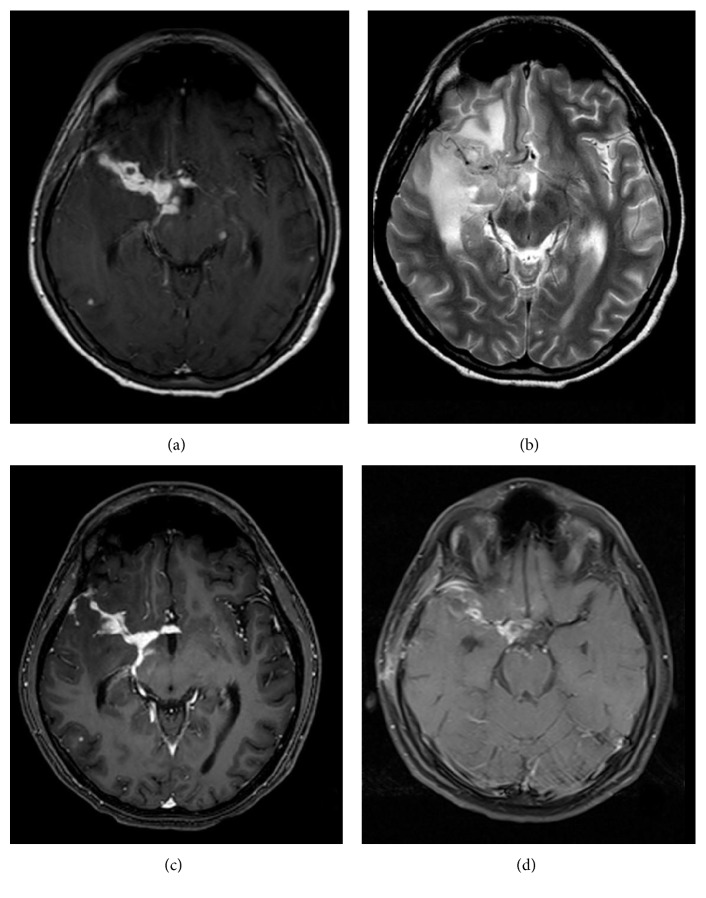
Contrasted T1A (a) and T2A (b) images 60 days after the diagnosis, contrasted T1A (c) image of 75 days later, and contrasted T1A (d) image of 90 days after the diagnosis. Considerable progression of the basal meningitis and parenchymal tuberculomas was seen after the 60th day instead of tuberculosis regression (a, b). A contrast is seen in the scattered parenchymal tuberculoma (a). After this examination, the patient began taking a steroid. There is a reduction of the basal meningitis and tuberculoma at the contrasted T1A image after 15 days on the steroid treatment. (c) A disappearance of tuberculomas, regression of contrast involvement, and exudate on the right sylvian fissure are seen on the contrasted T1A image 30 days after the steroid treatment.

**Figure 4:**
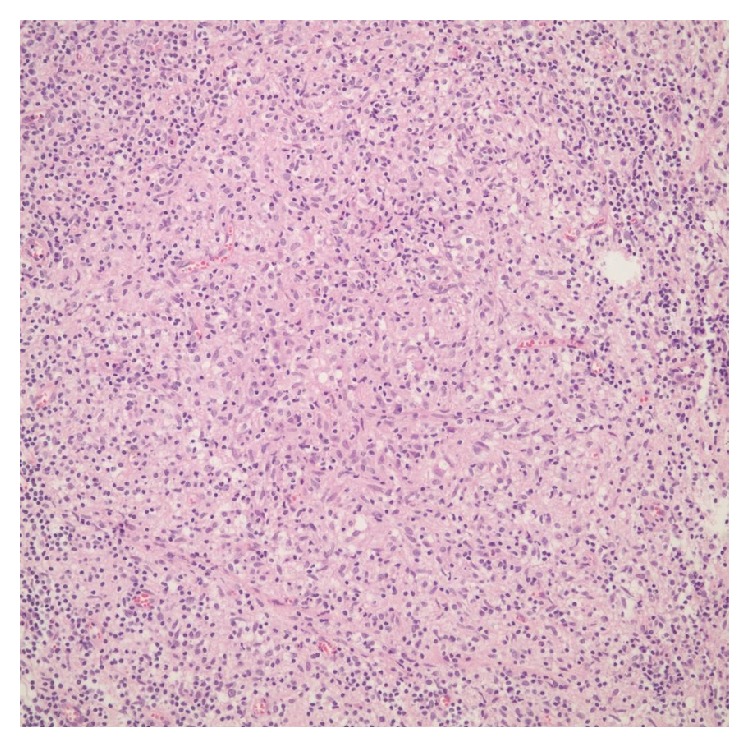
H&E, 200x. The lymphohistiocytic reaction and abortive granuloma structures.

One week later, after 45 days of the anti-TB treatment, the patient was admitted into our hospital because of a severe headache. In the initial evaluation, the patient was conscious, cooperative, and oriented, and his vitals parameters were normal. A Bacillus Calmette-Guerin (BCG) scar was present. He had right-central facial paralysis and strength loss, specifically on the left half of the body. Dysmetria, tremor, and prominent loss of fine motor coordination were all present in the upper left extremity. Other physical examination findings were normal. In laboratory examinations, complete blood count (CBC) test, C-reactive protein (CRP), and erythrocyte sedimentation rate were in normal limits. Additionally, the test for antibodies against the human immunodeficiency virus (HIV) was negative and biochemical analysis of serum was normal. Cerebrospinal fluid (CSF) samples were microscopically normal and CSF opening pressure was 16 cm of water, the CSF protein level was 198 mg/dL (15–40), and the CSF glucose level was 46 mg/dL (60–80) (with a simultaneous blood glucose level of 72 mg/dL). No organisms were recovered in aerobic CSF culture. There was not any evidence for TB in the anteroposterior chest X-ray. MRI findings of the cerebral and cerebellar regions were a wide edema area in the frontotemporal white matter and a chronic infarct sequel area shown in the leptomeningeal region. In comparison with previous MRI, it was shown that the same lesion had clearly enlarged (Figures 1, 2, and 3). Moxifloxacin and clarithromycin were added to the isoniazid, rifampicin, ethambutol, and pyrazinamide treatment on account of the possible existence of resistant* M. tuberculosis* such as father's pattern. On the other hand, oral prednisolone (1 mg/kg/day) was added to the management protocol because of the prospect of the paradoxical response. A brain biopsy was performed to confirm the diagnosis and a lymphohistiocytic reaction, giant cell formation, and a granulomatous response, which is characterized by abortive granulomas, but does not include caseous necrosis, are all seen in the microscopic examination of the tissue (Figure 4). Polymerase chain reaction (PCR) of the brain tissue was positive for* M. tuberculosis*. Tissue cultures were negative in terms of aerobic microorganism, fungus, and* M. tuberculosis*. The primary immunologic workup including lymphocyte subgroups, immunoglobulins, and slide nitroblue tetrazolium (NBT) test was normal as well as interleukin- (IL-) 12 receptor B1 expression and interferon (IFN) gamma functions. A decreasing size of tuberculoma was detected in the control MRI. The patient's complaints were minimized under the medication of isoniazid, ethambutol, rifampicin, moxifloxacin, and clarithromycin. The patient participated in physiotherapy and rehabilitation program in addition to symptomatic treatments for spasticity and was discharged after 45 days of hospitalization. Tuberculoma was not revealed in the cranial MRI of the patient one month later. Steroid treatment was decreased at 8 weeks and interrupted at 12 weeks after discharge.

## 3. Discussion

In 1955, a paradoxical response under TB treatment was first identified by Choremis and his friends on lung graph imaging results of a child who was being treated for TB [5]. In 1974, Thrush and Barwick, for the first time, documented paradoxical reaction in a patient with CNS TB, who had multiple tuberculomas and developed a new tuberculoma during treatment with anti-TB drugs [6]. In 1980, Lees and coworkers described the first report of paradoxical reaction of TB meningitis in 2 female patients, who paradoxically developed multiple cerebral tuberculoma and basal arachnoiditis [7]. There is limited pediatric data about the paradoxical response, which confuses physicians on the accuracy of the diagnosis leading to invasive diagnostic modalities such as brain biopsy and being forced to add unnecessary antimicrobial new drugs as in our patient. Understanding of the clinical nature of this response will let us avoid adverse outcomes of diagnostic and therapeutic modalities. Therefore, we share the hope that the increase in reports of the concept of paradoxic response which has been made possible by the awareness of pediatricians will throw light on appropriate management of children with TB.

Paradoxical deterioration mostly occurs in patients with extrapulmonary and disseminated TB, like miliary TB and TB meningitis. The CNS and the respiratory system remain the most common sites of involvement during paradoxical deterioration reported in literature. For the CNS manifestations, patients may have headache, mental confusion, focal seizures, cranial nerve palsies, and cortical signs such as hemiparesis, paraparesis, and hemianaesthesia as a result of the enlargement or development of intracranial tuberculomas and hydrocephalus [8]. Our patient presented with severe headache and hemiparesis caused by enlargement of the CNS lesion too. The reason why a paradoxical response occurs is not known exactly. Various hypotheses have been suggested to explain this unusual phenomenon. One of them is that this response occurs as a result of decreased penetration of antitubercular drugs into the brain. Restoration of blood brain barrier with appropriate treatment is proposed to result in reactivation of latent foci. However, this hypothesis cannot explain the development or enlargement of intracranial tuberculoma which is treated with isoniazid and pyrazinamide, both of which freely cross the blood brain barrier in the absence of inflamed meninges. Enlargement of lymph nodes (which do not have the barrier like blood brain barrier) in patients on anti-TB therapy further goes against the hypothesis. The most likely explanation for paradoxical response is interplay between host's immune response and the direct effect of mycobacterial products [4]. A paradoxical response can occur 3–12 weeks after the initiation of a TB treatment; however, it can take as long as 18 months [9]. It was seen in our patient 8 weeks after the treatment with several CNS symptoms.

A paradoxical response is commonly seen in 6–30% of patients being treated for TB [8], particularly in adult and immunocompromised patients. This situation is seen very rarely in children and the earliest age at which it has occurred was 21-day-old child who was treated for congenital lung TB [10]. Although few adult paradoxical responses are reported in Turkey, a paradoxical reaction has never been reported in children who were diagnosed with TB meningitis. The aforementioned case is one of the rarely seen and reported cases of a child having a paradoxical reaction. Furthermore, although the reported rate of the paradoxical response in patients with HIV infection is 35%, this rate is lower than 5% in TB patients with normal immune systems [10]. HIV was negative in our case and primary immunologic workup as well as IL-12 receptor B1 expression test and IFN-gamma functions were normal as well. Our patient did not have a mendelian susceptibility to the mycobacterial diseases. Additionally, some stated risk factors for the paradoxical reaction are anemia, hypoalbuminemia, and lymphopenia [1]. There was no mentioned risk factors in our patient. It is not necessary to change or stop anti-TB treatments, when a paradoxical response develops. Also, 95% of mycobacteria are sensitive to the treatments [10], but resistance to anti-TB drugs is still important, especially in our country where proliferation of resistant TB strains is an ongoing process [1].

Paradoxical reactions are treated with a systemic corticosteroid and/or surgery.

Corticosteroids decrease intracranial pressure, which is helpful in diminishing any of the disease's neurological symptoms [3]. Systemic corticosteroid treatment was added to the treatment of our patient with a good clinical response. A ventriculoperitoneal shunt implementation is a surgical alternative if a medical treatment fails; however, surgical treatment is not needed for our case.

Despite appropriate treatment, the cause of the paradoxical response in TB patients is not clear. Even though his father was diagnosed with lung TB caused by a resistant form, a prophylaxis for TB was not given to our case. Firstly, it was thought that the patient would not respond to the medication because of the suspicion of the possible resistant infection as in his father's case. As a result, new antimicrobials were added to the treatment. Subsequently, a very invasive procedure such as brain biopsy was performed to confirm diagnosis due to the fact that there was no enough improvement with those new drugs. Consequently, paradoxic response may lead to a confusion in the management of the TB disease. Thus, it is clear that understanding the nature and recognizing the paradoxic response will help the clinical practice of the pediatrician to avoid unnecessary management strategies.
